# Cardiac remodeling in ambitious endurance-trained amateur athletes older than 50 years–an observational study

**DOI:** 10.1371/journal.pone.0266951

**Published:** 2022-04-12

**Authors:** Daniel Dalos, Theresa Dachs, Constantin Gatterer, Matthias Schneider, Thomas Binder, Diana Bonderman, Christian Hengstenberg, Simon Panzer, Stefan Aschauer

**Affiliations:** 1 Department of Internal Medicine II, Division of Cardiology, Medical University of Vienna, Vienna, Austria; 2 Department of Blood Group Serology and Transfusion Medicine, Medical University of Vienna, Vienna, Austria; The Open University, UNITED KINGDOM

## Abstract

**Background:**

Data on cardiac remodeling in veteran athletes are conflicting but of clinical importance.

**Methods:**

Sixty-nine clinically stable and healthy individuals >50 years were identified (median 55 (IQR 52–64), 26% female). Echocardiographic features were identified in individuals, who have performed endurance sports at 70% of their maximum heart rate for at least 1 hour 3 times/ week over the previous 5 years.

**Results:**

Median training time in all participants was 6 hours per week. Therefore, based on these 6 hours of weekly training, participants were grouped into 45 ambitious endurance-trained amateur athletes (EAA) and 24 recreationally active endurance-trained athletes (RAP) training ≥6 hours (6–10) and <6 hours (3.5–5), respectively. Left ventricular (LV) diameters were slightly larger in EAA than in RAP (27 mm/m^2^ (25–28) vs. 25 mm/m^2^ (24–27), p = 0.023) and EAA showed preserved diastolic function (p = 0.028) with lower E/E’ ratio (7 (6–9) vs. 9 (7–10), p = 0.039). Interventricular septal thickness and relative wall thickness ratio were similar. Global right ventricular and LV strain were similar, but left atrial (LA) reservoir strain was higher in EAA than in RAP (27% (22–34) vs. 20% (15–29), p = 0.002).

**Conclusions:**

Endurance training in healthy athletes >50 years is not associated with chamber dilatation or LV hypertrophy. A weekly training duration of ≥6 hours seems beneficial to preserve diastolic function associated with an increased LA reservoir function.

## Introduction

A century of research was focused on accommodation of the athletes’ hearts to repetitive exercise-induced increased physiological demand [1–3]. Subtle myocardial adjustments can nowadays be detected by echocardiography, summarized in excellent reviews and meta-analyses [3–7].

There might be differences between endurance versus resistance exercise-induced cardiac remodeling [6]. Nowadays, top-class athletes spend substantial time on resistance exercises to build up their muscular power. However, endurance training remains the prerequisite in the majority of resistance and skill sports. Thus, Beaumont et al. described only after categorization, as proposed by Mitchell et al. [8], subtle differences emerge between static-resistance and dynamic–endurance demands, predominantly in twist mechanics [9].

Currently accepted features of the endurance trained athlete’s heart include biventricular and biatrial dilatation, ventricular hypertrophy, and normal or slightly reduced resting biventricular systolic function [3]. Recent publications have focused on right ventricular (RV) remodeling [10, 11]. Sex, body-surface area and history of high-level endurance training were determinants of the right atrial size [12], and age determines a variety of remodeling adaptations [13–15]. Functionally, the heart’s ability for remodeling facilitates stroke volume augmentation and thus increases cardiac output reserve during exercise.

All the aforementioned studies have been performed on athletes younger than 50 years of age, many including athletes even younger than 40 years. Data from older individuals have been comprehensively reviewed by Beaumont et al. [16] and complemented by additional studies [17–19]. Others described rather small cohorts [16, 18], or cohorts of mixed young and older individuals [11, 20–23]. In addition to the remodeling capacities, Bohm et al. addressed the question if too ambitious engagement in endurance sports in older adults is deleterious. No evidence for such an assumption was found [11]. Prompted by the aforementioned study, Wasfy and Baggish, emphasized the importance of distinguishing between healthful physiological adaptation to endurance exercise and its continuum to pathology, whereby the latter may differ from one individual to the next [24].

A physically active population in senior age in good physical condition devotes more and more time to regular physical exercise, often participating in aged-matched national or international competitions. They prefer endurance sports, like running, swimming, and biking. These sports are popular because they are, at least in part, associated with a low risk for injury and can be maintained further into advanced age. Due to their advanced age, this population may seek professional healthcare advice more regularly than younger individuals. Hence it is of great clinical relevance to explore potential changes in chamber dimension and function as well as adaptation in wall thickness in older adults in response to ambitious physical exertion. We hypothesized that, similar to young athletes’ hearts, endurance sport activities lead to cardiac transformations based on the weekly duration of exercise in adults aged older than 50 years. At advanced age, it is of utmost importance to differentiate between healthful physiological adaptation to endurance exercise and pathology.

## Methods

### Participants

This single-center, observational study was approved by the ethics committee of the Medical University of Vienna in accordance with the Declaration of Helsinki.

We defined the target population as individuals who are active in their sport discipline for at least 3 times per week, each time for at least one hour at a heart rate (HR) of 70% of their individual maximal HR. These inclusion criteria should allow revealing changes in a recreational active population (RAP) compared to ambitious endurance-trained amateur athletes (EAA). Healthy individuals, members of two different sporting-clubs, were actively invited to participate. They were all Caucasians from the Viennese urban area and provided written informed consent for participation. All fulfilled the following criteria for eligibility to enter the study: age >50 years, endurance sports at a HR of 70% of their maximal HR for at least 3 times per week for at least 1 hour. A prerequisite for participation was the fulfillment of these criteria already for at least the previous 5 years. Aiming to include athletes in their advanced adulthood, we excluded younger athletes in order to form a coherent cohort. Only 20 EAA athletes have started with regular running at the age of 40 years or later, with less than 6 hours per week, and consecutively advanced to their current training schedule. All other EAA participants have been actively running or swimming already since early adulthood (age 20 to 35 years).

In the RAP cohort, 16 participants have started regular sporting activity after the age of 35 years, the others started already earlier. In addition, these participants have started with considerably less training time, which was increased gradually. All EAA participants reported to participate regularly in national and international competitions three or more times per year at least during the previous 5 years. All of the RAP cohort participated at least once a year at local and national competitions, except of 2 runners.

All participants reported interval training as a regular part of their endurance training, achieving 70% of the maximum heart rate [25]. All EAA participants included anaerobic intermittent high-intensity exercise once or twice a month, while high-intensity exercise was not on a regular schedule in the RAP cohort.

**Fig 1:**
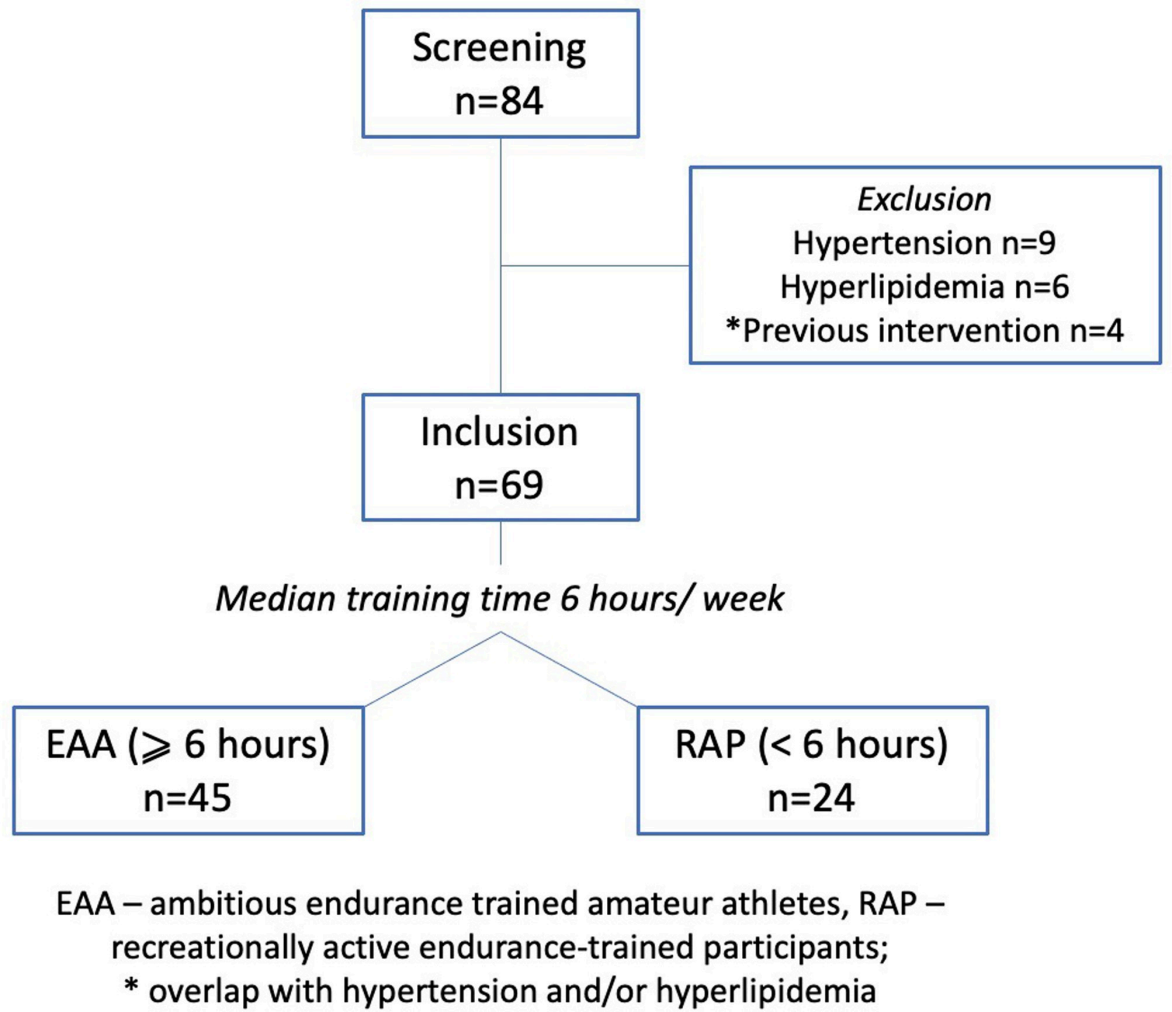
Flow-chart of the inclusion process.

All participants were taking graded exercise tests to their limit once per year, to confirm the absence of any apparent cardiovascular (CV) disease and to be eligible for competitions. All participants answered a questionnaire providing the following information: previous diseases or surgical procedures, past or current smoking, current medication, inherited or acquired abnormalities of their upper body, previous physical activity, other than the current one, duration of active lifestyle, type of current main sport, and duration and number of sessions per week. Based on this questionnaire prior to the current investigation, we excluded individuals with any medical condition, which may affect the CV system, like hypertension or dyslipidemia, or previous interventions like percutaneous coronary intervention or cardiac surgery [25] (*Fig 1*).

Baseline demographical data such as height, weight, body mass index, heart rate and blood pressure in the supine position were recorded.

### Laboratory analysis

Complete blood count and blood chemistry were performed. Serum N-terminal pro brain natriuretic peptide (NT-proBNP) was measured with an immunological test (Elecsys^®^ Systems, Roche Diagnostics, Rotkreuz, Switzerland).

### Echocardiography

Transthoracic echocardiography (TTE) examinations were performed by DD and SA using GE Vivid E9 and E95 scanners (GE Healthcare, Wauwatsa, WI, USA). All measurements were performed adhering to the guidelines of the European Association of Cardiovascular Imaging and the American Society of Echocardiography [26]. Strain analysis was performed by speckle tracking technique using GE EchoPAC software and images were recorded according to the recommendations given by an expert consensus statement [27].

Left ventricular (LV) ejection fraction (EF) was assessed using the biplane Simpson´s method and global longitudinal LV strain was performed according to the recent recommendations [26]. Pulsed-wave Doppler was performed to obtain the early (E) to late (A) ventricular filling velocities. E´ (early diastolic mitral annular velocity) was assessed at the septal and lateral side of the mitral annulus with Tissue Doppler Imaging and averaged to calculate E/E’. Relative wall thickness ratio was calculated using the following formula: two times wall thickness of the posterior wall divided by LV end-diastolic diameter [28]. LV strain recording was done in apical 4-, 2- and 3-chamber views defining 6 myocardial segments in each view. These segments were then averaged resulting in strain values for basal, mid and apical portions.

RV function (RVF) was assessed by integrating visual assessment of contractility of the RV outflow tract, RV apex, and interventricular septum from different views. In addition, RV strain was calculated from the free RV wall by tracking basal, mid and apical wall segments, which were then averaged for global longitudinal RV strain. Furthermore, tricuspid annulus plane systolic excursion (TAPSE) as well as tissue doppler imaging (TDI) from the tricuspid annulus were performed [26]. Tricuspid regurgitation (TR) as well as TR velocity were quantified according to recent recommendations [29].

Using the aforementioned parameters, diastolic function was quantified according to the recent guidelines [26].

*Normal diastolic function*: E/A ≥ 0.8, E/E’ < 10, TR velocity < 2.8m/sec,
*Impaired relaxation (grade I)*: E/A ≤ 0.8, E/E’ < 10, TR velocity < 2.8m/sec,
*Pseudonormal filling pattern (grade II)*: E/A > 0.8 - < 2.0, E/E‘ 10–14, TR velocity > 2.8m/sec,
*Restrictive filling pattern (grade III)*: E/A > 2.0, E/E’ > 14, TR velocity > 2.8m/sec.

**Fig 2:**
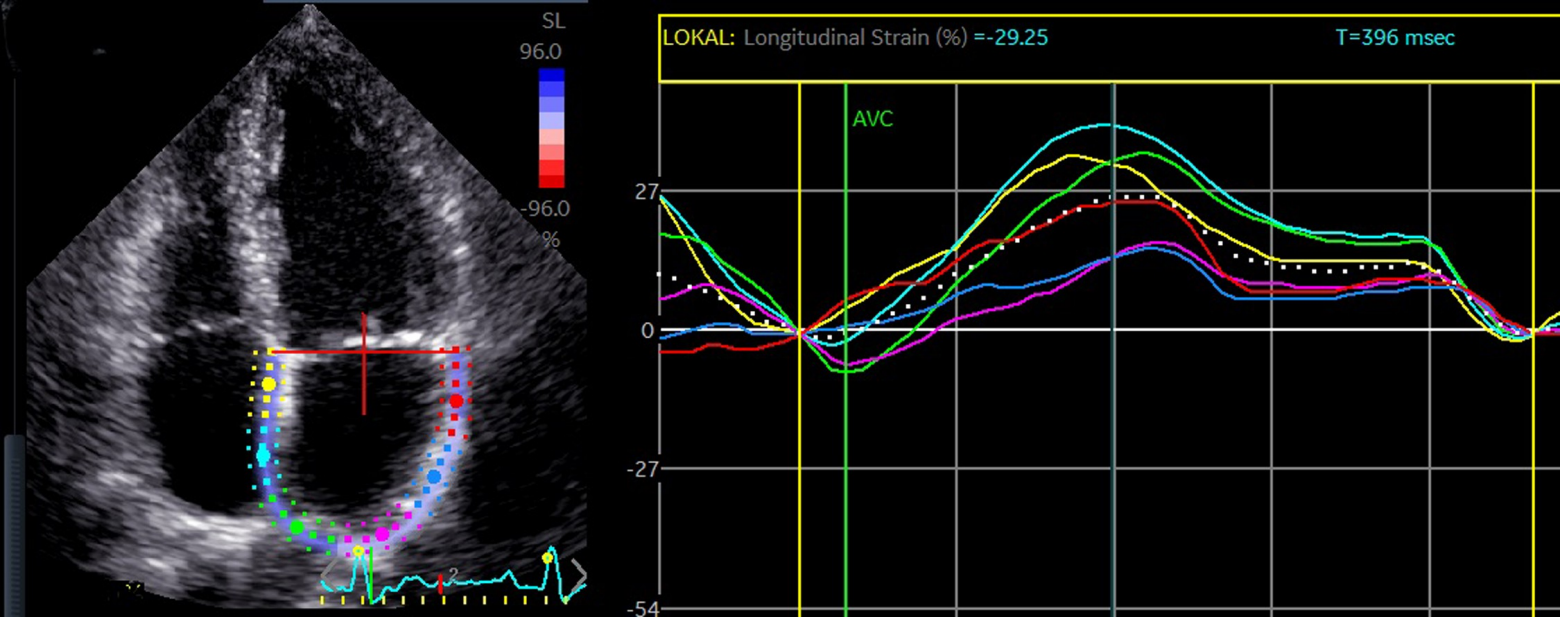
Two-dimensional left atrial (LA) reservoir strain from apical 4-chamber view. The current proband shows a LA strain function of 26%.

Additionally, left atrial (LA) strain analysis was performed [30] (*Fig 2*). Images were acquired in 4- and 2-chamber views with optimized image quality in order to trace LA endocardial border. LA reservoir strain was defined as the difference between the first negative peak (contractile strain) and the maximum positive peak (conduit strain) and was averaged in all segments as well as both views [27, 30]. TTE measurements were performed by DD and SA with a good intraclass correlation coefficient of 0.91.

### Statistical analysis

Statistical analyses were performed using STATA 11 (Stata Corp LP, College Station, TX, USA). Data are shown as median and interquartile range (IQR) or frequencies and percentages. Categorial variables were compared using chi-squared test or Fisher’s exact test and the Wilcoxon rank sum test was applied for continuous variables. A two-sided P-value <0.05 was considered significant.

## Results

Between November 2018 and May 2019, 69 individuals fulfilled all of the above listed criteria and were included in the analyses. Their median age was 55 years (IQR 52–64) and 26% were female. The study population included long-distance runners (65%), swimmers (23%), and triathletes (12%) with a median duration of endurance training of 6 hours per week. We therefore defined a group of EAA with 45 participants training ≥ 6 hours per week (IQR 6–10 hours) and a control group (RAP) including 24 participants with an exercise duration of < 6 hours per week (IQR 3.5–5 hours). EAAs were runners (63%), swimmers (29%) and triathletes (8%); in RAP, 67% were runners, 20% swimmers and 13% triathletes. Groups did not differ regarding age, sex or body mass index (*Table 1*).

**Table 1:** Baseline characteristics.

	*All participants*	*EAA*	*RAP*	*p-value*
	*(n = 69)*	*(n = 45)*	*(n = 24)*	
**Clinical characteristics**				
Age, years	57 (52–64)	55 (52–63)	57 (54–66)	0.684
Female sex, n (%)	18 (26.1)	14 (31.0)	4 (16.7)	0.199
Height, cm	176 (174–177)	175 (173–177)	178 (174–181)	0.164
Weight, kg	77 (74–80)	74 (71–78)	81 (75–86)	0.165
Body mass index, kg/m2	24 (23–26)	24 (22–26)	25 (23–27)	0.086
Heart rate, bpm	61 (59–63)	60 (57–63)	63 (59–67)	0.205
Systolic blood pressure, mmHg	127 (124–129)	128 (125–130)	125 (120–130)	0.260
Diastolic blood pressure, mmHg	83 (82–84)	83 (82–85)	83 (81–85)	0.825
Alcohol intake, n (%)	51 (73)	30 (67)	21 (88)	0.061
Current smoker, n (%)	6 (9)	3 (7)	3 (13)	0.670
Exercise per week, hours	6 (4.5–8)	7 (6–10)	4 (3.5–5)	< 0.001
**Laboratory**				
Hemoglobin, g/dl	14.7 (14.1–15.2)	14.6 (13.8–15.2)	15.0 (14.4–15.3)	0.200
Hematokrit, %	42 (41–43)	42 (40–43)	42 (41–44)	0.265
HbA1c, %	5.4 (5.2–5.5)	5.4 (5.2–5.5)	5.4 (5.2–5.6)	0.967
NT-proBNP, pg/ml	38 (17–76)	43 (15–74)	33 (18–94)	0.378

Continuous variables are given as medians and inter-quartile ranges. Counts are given as numbers and percentages. EAA–ambitious endurance-trained amateur athletes, NT-proBNP–N-terminal pro brain natriuretic peptide, RAP–recreationally active endurance-trained participants.

Routine laboratory parameters such as hemoglobin or HbA1c were within normal ranges in both groups, as well as NT-proBNP values, excluding any advanced CV disorder such as significant valvular heart disease (*Table 1*).

Echocardiographic analyses (*Table 2*) revealed that EAA had smaller right and left atrial diameters compared to RAP (p = 0.045 and p = 0.039, respectively), although these differences were not apparent when diameters were indexed to body surface area. Additionally, no differences were observed in LA volumes (p = 0.873), but LV end-diastolic diameters were slightly larger in EAA (p = 0.023) accompanied by a minimally lower ejection fraction compared to RAP (p = 0.017). Mild TR was present in 26% of all participants without any differences between groups but TR velocities were higher in EAA than in RAP (p = 0.031). Both groups showed similar interventricular septal thickness as well as relative wall thickness ratio.

**Table 2:** Echocardiographic analyses.

	*All participants*	*EAA*	*RAP*	*p-value*
	*(n = 69)*	*(n = 45)*	*(n = 24)*	
RA diameter, mm	53 (49–55)	53 (49–55)	53 (52–58)	**0.045**
RA diameter-Index, mm/m^2^ BSA	27 (26–29)	27 (25–29)	27 (25–30)	0.989
RVEDD, mm	34 (31–37)	34 (31–37)	35 (32–38)	0.336
RVEDD-Index, mm/m^2^ BSA	18 (16–19)	18 (16–20)	17 (16–19)	0.417
TAPSE, mm	26 (23–29)	25 (23–29)	27 (25–29)	0.200
RV-TDI, m/sec	0.15 (0.14–0.16)	0.15 (0.13–0.16)	0.16 (0.15–0.16)	0.064
Mild TR, n (%)	18 (26)	12 (27)	6 (25)	0.779
TR-velocity, m/sec	2.5 (2.3–2.8)	2.5 (2.4–2.8)	2.3 (2.2–2.5)	**0.031**
PAP systolic, mmHg	30 (26–36)	30 (28–36)	25 (20–30)	0.141
LA diameter, mm	53 (49–55)	52 (49–55)	53 (52–58)	**0.039**
LA diameter- Index, mm/m^2^ BSA	27 (26–29)	27 (25–29)	27 (26–30)	0.880
LA volume, ml	75 (64–82)	75 (63–82)	72 (65–90)	0.842
LA volume- Index, ml/m^2^ BSA	39 (33–44)	39 (34–44)	37 (33–45)	0.873
LVEDD, mm	50 (47–52)	50 (47–53)	50 (47–52)	0.648
LVEDD-Index, mm/m^2^ BSA	27 (25–28)	27 (25–28)	25 (24–27)	**0.023**
Ejection fraction, %	59 (57–63)	59 (56–61)	63 (58–64)	**0.017**
IVS, mm	11 (10–12)	11 (10–12)	11 (10–12)	0.427
Relative wall thickness ratio	0.5 (0.5–0.6)	0.5 (0.5–0.6)	0.6 (0.5–0.6)	0.409
Mild MR, n (%)	23 (33)	12 (27)	11 (46)	0.108
E/E’	7 (6–9)	7 (6–9)	9 (7–10)	**0.039**
Diastolic function				**0.028**
Normal function, n (%)	39 (57)	28 (62)	8 (33)	
Impaired relaxation, n (%)	27 (39)	17 (38)	13 (54)	
Pseudonormal filling, n (%)	1 (1)	0	1 (4)	
Restrictive filling, n (%)	2 (3)	0	2 (8)	
Global RV strain, %	-26 (-27–-21)	-25 (-28–-22)	-26 (-27–-21)	0.864
Basal RV strain, %	-23 (-26–-20)	-23 (-26–-20)	-24 (-26–-20)	0.543
Mid RV strain, %	-26 (-27–- 21)	-25 (-28–-21)	-26 (-27–-21)	0.883
Apical RV strain, %	-27 (-30–-21)	-27 (-30–-21)	-28 (-31–-25)	0.514
Global LV strain, %	-19 (-21–-18)	-19 (-21–-17)	-19 (-21–-18)	0.445
Basal LV strain, %	-16 (-17–-14)	-16 (-17–-14)	-16 (-17–-15)	0.714
Mid LV strain, %	-18 (-19–-17)	-18 (-19–-16)	-18 (-20–-17)	0.143
Apical LV strain, %	-25 (-27–-21)	-24 (-28–-21)	-25 (-27–-22)	0.803
LA reservoir strain, %	25 (20–31)	27 (22–34)	20 (15–29)	**0.004**

Continuous variables are given as medians and inter-quartile ranges. Counts are given as numbers and percentages. EAA–ambitious endurance-trained amateur athletes, BSA–body surface area, E–early mitral inflow velocity, E’–early diastolic mitral annular velocity, IVS–interventricular septal thickness, LA–left atrium, LV–left ventricular, LVEDD–left ventricular end-diastolic diameter, MR–mitral regurgitation, PAP–pulmonary artery pressure, RA–right atrium, RAP–recreationally active endurance-trained participants, RV–right ventricular, RVEDD–right ventricular end-diastolic diameter, RV–TDI–right ventricular tissue doppler imaging, TAPSE–tricuspid annular plane systolic excursion, TR–tricuspid regurgitation, TR-velocity–tricuspid regurgitation velocity.

Diastolic function was different between the groups (p = 0.028). In EAA, 62% of participants displayed a normal diastolic function compared to 33% in RAP. An impaired relaxation pattern was found in 38% in EAA and in 54% in RAP. One subject in RAP showed pseudonormal filling and two individuals in RAP had restrictive filling patterns. Moreover, E/E’ ratio was significantly lower in EAA (p = 0.039).

Global left and right ventricular systolic functions were similar in all participants (p>0.05).

Speckle-tracking analysis was similar in both groups with respect to global longitudinal LV and RV strain measurements, specific apical, mid, and apical segments (*Table 2*). Interestingly, EAA showed superior reservoir function in global LA strain as compared to RAP (27% (IQR 22–34) vs. 20% (IQR 15–29), p = 0.002).

## Discussion

In this cross-sectional analysis, we observed a significant difference in the LV diastolic function and the LA reservoir function in EAA compared to RAP. These results suggest that cardiac remodeling in response to the increased physiologic demand persists in advanced age.

Overall, there is some controversy on cardiac remodeling in advanced age [16]. In order to shed more light on cardiac changes in physically active individuals we stratified the total population into two groups according to the median time of weekly sports activity, namely recreational activity, and ambitious amateur athletes. We cannot exclude the possibility that a different classification would have disclosed less differences, e.g. taking 4 hours of training per week as a cut-off. However, a prerequisite to participate in this study was exercise for at least 3 hours per week at a HR of 70% of the individual maximal HR, which may have led to the observed results if taking the time for a warm-up and cool-down phase into account. All participants included interval training into their regular routine. This type of exercise is known to provide a greater challenge to the cardiopulmonary, peripheral, and metabolic systems and results in a more efficient training effect [25].

We may assume that a 6 hours weekly training routine represents the real-life scenario in older individuals who ambitiously prepare for competitions next to their obligations for their family and profession.

### Cardiac remodeling

It shall be kept in mind, that adults who are devoted to regular sport activities into later adulthood have most likely been active at a young age, too. Therefore, remodeling of their heart may have occurred earlier in life. In a follow-up study 12 years after a baseline cardiac assessment of veteran endurance runners aged 56 to 83 years, interventricular septum and LV wall hypertrophy was seen in 53% (10 of nineteen individuals) [31]. However, this hypertrophic adaptation had already been recorded at the initial examination [31]. Some participants in this study, at least to some extent, suffered from arterial hypertension, which can lead to LV hypertrophy on its own. Nevertheless, these changes persisted into later in life as long as exercise was continuously performed, without any concomitant LV dilatation, which may occur in young athletes or middle-aged individuals with moderate physical activity [23, 31]. This is in line with our findings, demonstrating the absence of any LV hypertrophy or a significant LV dilatation. Therefore, alterations in interventricular septal thickness in healthy very active older veterans cannot be solely attributed to endurance training and may need further medical attention.

Similar to the LV, the RV has also been described to undergo remodeling as a consequence to extensive training in young elite athletes. Previous studies showed RV enlargement and reduced myocardial function in young elite athletes [10]. All our participants, however, showed normal RV size and function.

### Diastolic function

There is still controversy on the effect of endurance training on diastolic function. A variety of small studies suggested an augmented diastolic filling in young endurance athletes [3, 32], whereas others did not find differences in diastolic function parameters compared with controls [33]. In general, diastolic function has previously been shown to deteriorate with progressing age, which is mainly responsible for the increasing prevalence of heart failure (HF) with preserved ejection fraction in older adults [34]. In addition, impaired cardiorespiratory fitness in advanced age may further contribute to a gradual deterioration of diastolic function. This hypothesis has been the focus of a variety of studies including small numbers of participants. Douglas et al. investigating 45 athletes suggested that ultra-endurance training was an effective stimulus in shaping the LV structure and, thus, in preserving diastolic function [35]. In 1993, Levy and coworkers investigated 14 men, aged 60 to 82 years, and found that 6 months of endurance exercise training enhanced early diastolic filling and reduced resting atrial filling rates [32]. After the turn of the century, an additional study with a relatively small sample size confirmed the beneficial effects of prolonged, sustained endurance training on LV compliance which may prevent HF in advanced age [36]. More recently, Santoro examined 125 amateur swimmers of different ages and showed that regular aerobic exercise was helpful in minimizing age-related alterations on diastolic function [37]. In addition, a study performed in a rat model also demonstrated an attenuation of age-associated diastolic dysfunction due to endurance training, which might be attributed to the positive effects on coronary microvascular function [38].

However, the study by Fleg et al. revealed diastolic filling rates in 16 older male endurance athletes which were similar to their sedentary peers [39]. Furthermore, Teske et al. evaluated 53 veteran athletes and did not find beneficial effects of training on age-related diastolic impairments when compared to controls [13]. It is noteworthy that these individuals were slightly younger (inclusion criterion: 40 years or older) than in the present study, and, interestingly, E/E’ ratios were lower in elite athletes compared to regular athletes, as well as in veteran athletes when compared to age-matched controls [13]. Although these differences did not reach statistical significance, they are in line with our findings of lower E/E’ ratios in EAA. Despite the fact that endurance training did not result in changes in several doppler measurements, Teske and his coworkers report that global diastolic function seems to be preserved in veteran athletes [13], which is in line by the findings reported more recently by Banks et al. [17]. The review by Beaumont et al. further supports the assumption of a superior diastolic function in endurance trained athletes [16].

Another interesting finding of our study is that individuals with a workout duration of more than 6 hours per week show an increased LA reservoir strain compared to those who exercise less. A decrease in LA reservoir strain has recently been identified as an early marker of diastolic dysfunction in patients with arterial hypertension and atrial fibrillation, even in the absence of diastolic doppler measurement abnormalities [30]. Recently, a meta-analysis of more than 400 young, predominately male, elite athletes has suggested a reduced global LA longitudinal strain, resembling LA reservoir function, compared with age-matched controls [40], which might represent a physiological adjustment in response to the training frequency and intensity in young athletes. Our results suggest that the LA reservoir strain might recover over several years of ambitious endurance training in veterans, which is of enormous clinical relevance because a reduced LA strain has previously been described as an independent predictor of HF hospitalizations and all-cause mortality [41].

### Limitations

First, we did not recruit a control group of sedentary individuals, because of the difficulty to find an age- and sex-matched cohort without CV disease. However, as the differences between RAPs and EAAs are already apparent, we can assume even larger differences between our cohort of EAA and a sedentary population of the same age.

Second, duration and intensity of endurance training were self-reported and therefore no standardized workout regime was applied. Also, peak VO2 values would have been beneficial to assess the individuals’ fitness and the relation to remodeling. However, participants underwent yearly graded treadmill testing to their limit and recorded their workout regularly.

Third, a selection bias cannot be excluded as our cohort consists of healthy individuals and athletes with pre-existing, probably performance-limiting, CV diseases were not included in this trial.

Fourth, due to the cross-sectional design of this study a causal relationship between the effects of training and cardiac remodeling cannot be assessed.

Finally, this cohort was too small to differentiate between the effects of training modalities, like running-cycling-swimming. Particularly in younger individuals, training in a supine position, like swimming, affects exercise-induced cardiac remodeling differently than that in an up-ward position, like running [42, 43]. Furthermore, short-distance competitive running or swimming requires a different exercise schedule compared to long-distance. The participants in this study did not base their training on an aimed competition distance. Rather, non-differentiating interval training was the regular part of the endurance training.

## Conclusion

Cardiac remodeling in ambitious endurance-trained amateur athletes older than 50 years is not related to significant atrial or ventricular dilatation nor relevant LV hypertrophy. Prolonged training duration (e.g. ≥ 6 hours per week) seems to maintain diastolic function accompanied by an increased LA reservoir function in healthy individuals. These findings shall assist in defining healthful physiological adaptation to endurance exercise and pathology.

## Supporting information

S1 Dataset (PDF)
