# Supplementary material for: Cardiac remodeling in ambitious endurance-trained amateur athletes older than 50 years–an observational study
Source: PLoS One. 2022 Apr 12;17(4):e0266951. doi: 10.1371/journal.pone.0266951 (PMC9004772; doi:10.1371/journal.pone.0266951)
Supplement: S1 Dataset — (PDF) [file pone.0266951.s001.pdf]

id	age	sex	height	weight	bmi	rauchen
1	55	2	172	60	20,3	0
2	55	1	168	60	21,26	0
3	52	1	180	67	20,68	0
4	67	1	184	105	31,01	0
5	50	1	187	84	23,74	0
6	60	2	161	55	21,22	ja
8	51	1	176	76	24,54	ja
9	50	1	177	73	23,3	0
10	58	1	172	75	25,35	0
11	57	2	168	76	26,93	ja
12	65	1	180	75	23,15	0
13	58	1	180	97	29,94	0
14	66	1	174	78	25,76	0
16	60	1	172	94	31,77	0
17	50	1	182	87	26,26	0
18	55	1	180	80	24,69	0
19	52	1	179	74	23,1	0
20	57	1	190	95	26,32	0
21	63	2	168	66	23,38	0
22	48	2	170	62	21,45	0
23	76	1	171	69	23,6	0
24	53	1	182	95	28,68	0
25	74	1	176	80	25,83	0
26	53	2	165	60	22,04	0
27	61	1	173	70	23,39	0
28	55	2	168	56	19,84	0
29	51	1	190	84	23,27	0
30	76	2	153	56	23,92	0
31	49	1	183	93	27,77	ja
32	54	1	187	73	20,88	0
33	70	1	167	74	26,53	0
34	52	1	185	78	22,79	0
35	59	2	172	59	19,94	0
36	50	2	172	62	20,96	0
37	52	1	185	79	23,08	0
38	62	1	178	79	24,9	0
39	54	1	181	74	22,6	0
40	68	1	183	100	29,9	0
41	58	1	181	84	25,6	0
42	64	1	186	105	30,4	0
43	53	1	185	78	22,8	0
44	59	2	164	69	25,7	0
45	67	1	180	74	22,8	0
46	50	1	190	105	29,1	0
47	82	1	165	71	26,1	0
48	50	1	182	78	23,5	0
49	66	1	170	73	25,3	0

50	55	1	169	72	25,2	0
51	71	1	169	77	27	0
52	50	2	170	73	25,3	0
53	53	2	172	63	21,3	0
54	50	1	180	85	26,2	0
55	67	1	179	73	22,8	0
56	53	1	180	78	24,1	ja
57	52	2	163	58	21,8	0
58	52	1	189	80	22,4	0
59	63	2	168	59	20,9	0
60	58	1	183	83	24,8	0
61	59	1	179	89	27,8	0
62	54	2	169	70	24,5	0
63	68	1	178	85	26,8	0
64	53	1	177	85	27,1	0
65	69	1	175	72	23,5	ja
66	67	1	172	70	23,7	0
67	50	2	168	84	29,76	0
68	51	2	161	50	19,3	ja
69	54	1	183	90	26,9	0
70	55	1	177	85	27,1	0
71	66	1	180	84	2,06	0

alkohol	hrsperweek	la	lv	ra	rv	aoasc
0	10	47	47	47	36	37
0	5	54	53	55	32	32
0	6	47	50	46	37	
0	4	67	49	63	38	34
0	8	60	55	62	32	33
0	4,5	44	47	42	24	28
1	6	54	44	54	34	
1	12	60	53	61	35	35
1	4	48	47	48	30	33
1	5	56	50	53	28	36
0	6	55	55	52	39	42
1	3	52	48	52	38	33
0	5	57	49	57	27	
1	12	54	50	57	35	37
0	3	52	44	53	40	
1	3,5	49	53	50	38	36
1	7,5	50	52	55	36	37
0	3,5	53	56	56	35	33
0	10	48	50	48	27	34
0	12,5	49	45	49	37	31
0	10	55	49	53	32	
0	10	50	50	50	36	38
1	3	58	57	59	33	38
1	10	57	45	57	35	30
1	6	50	50	50	36	35
0	3,5	49	44	49	31	33
1	6	51	54	51	45	35
1	6	42	44	42	28	32
0	8	55	49	55	31	34
1	5	49	50	56	34	34
1	7	55	54	53	37	38
1	25	56	55	53	28	36
0	7	49	46	48	27	33
0	6	49	49	49	25	34
1	5	54	48	52	42	34
1	6	49	49	49	34	35
1	12	53	54	53	37	34
1	5	75	56	75	34	36
1	6	49	47	49	30	36
1	3	62	47	63	38	36
0	3,5	61	50	66	36	29
0	10	43	47	43	31	28
1	4	50	49	49	34	32
0	6	54	53	54	31	38
1	6	53	48	53	27	36
1	10	54	54	54	37	40
0	4,7	58	52	58	26	31

0	8	57	50	58	37	40
0	6	64	50	61	37	30
1	7	54	51	56	37	30
0	10,5	55	50	55	28	
0	2,5	55	51	55	34	36
0	9	47	46	44	32	
1	5	55	43	53	36	35
1	6	47	48	47	31	36
1	7,5	48	55	51	36	32
0	6	52	51	50	32	36
0	6,5	56	52	55	38	37
1	6,5	55	49	55	29	35
1	7	53	47	53	33	
0	6	48	46	48	28	33
0	6	51	53	51	38	34
1	8	51	54	51	33	35
1	5	55	45	55	37	33
0	4	57	52	52	38	31
1	11	48	43	48	31	29
1	5	53	50	53	37	33
0	6	52	50	55	38	38
0	6	54	52	54	30	36

ivs	avmax	lvotmax	tivmax	ea	ee	lastrain
10	1,3		2,3	0,5	9	26,44
9	1,4	1,1		1	9	17,88
11	1,1			1,1	5	40,25
13	1,1	1,1			8	17,25
10	1,1	1,1		1,1	7	21,94
7	2,7	1,6		0,8	5	44,53
11	1,5	1,6		0,7	6	50,38
11	1,8	1,6	3,1	1	8	35,75
11	1,3			0,8	8	51,19
9	1,6	1	2,2	0,8	10	34,38
13	1,2	1	2,9	1,3	9	10,69
13	1,2	1,1	2,2	1	8	30,88
12	1,2	1,2	2,5	0,7	7	14,06
12	1,1			0,7	6	25,31
11	1,7	1,5	1,4	1,1	9	22,31
11	1,5	1,3		1,3	7	18,38
13	1,2	1,1		1,4	7	34,44
6	1,2	1		1	9	28,69
10	1,2			0,8	7	32,41
8	1,5		2,3	1,1	7	47,13
12	1,2		2,4	0,8	10	21,44
12	1,2			0,9	6	28,69
12	1,7	1,4	2,9	2,9	13	15,47
10	1,5		2,5	1,1	8	24,5
12	1,2	1,1	2,4	0,7	10	35
10	1	0,9	2,8	1,3	10	13,5
10	1,2		2,6	1,5	8	45,5
10	1,2	1,1		0,8	7	14,69
11	1,2	0,9	2,5	1,1	5	47,13
11	1,2	1	2,5	1,1	6	18,38
7	1,5	1,2	2,8	1,2	10	35,03
10	1,4	1,2	2,2	1,4	7	29,75
11	1,2	1,3	2,3	1	6	22,03
9	1,3	1,1	2,8	1	6	31,13
12	1,2	0,9	1,9		6	22,03
10	1,4	1,2		1	6	19,98
10	1,6	1,1	2,8	1,2	6	45,06
17	0,8	0,7		1,1	11	16,25
12	1,2	0,9		0,7	7	24,19
13	1,8	1,3		0,8	10	13,44
11	1,4	1,1		1,1	8	11,56
11	1,1	1		1,3	10	26,72
11	1,4	1,2		1,2	8	24,25
11	1,1	0,9	2,7	0,7	7	24
9	1,3	0,9			7	27,23
12	1,1	1,1		1,2	7	26,56
13	1,3			0,57	14	19,75

11	1,2	1	2,4	1,4	8	26,16
17	1,8		3		6	14,84
10	1,4	1,2	2,5	1,1	6	29,69
11	1,2	1,1		1,3	8	29,53
11	1,1	1		0,8	5	20,12
12	1,1	1,3		0,7	4	21,25
10	1,2	1,1			7	16,92
8	1,3	1,5	2,5	1,5	9	34,38
10					8	37,5
10	1,6	1,1	2,6	1,1	6	21,75
11	2,5			1,2	6	29,4
13	1,4	0,8	2,5	1	8	27
10	1	0,9	2,7	1,5	9	27,28
13	1,7	1,2		0,6	4	18,28
11	1,3		2,4	1,1	9	21,44
11	1,3	1,1	2,8	0,6	7	29,25
12	1,2		2,3	1,1	6	20,44
7	1,7	1,3		1,2	8	30,94
9	1,6	1,2	2,8	1,1	11	16,72
11	1,1	1,1	1,9	0,7	10	13,13
11	1,5	1,4		0,8	6	27
11	1,2	1		0,9	8	25

tdirv	tapse	lvf	diastole	rvf	mi	ms
0,13	30		0	0	0	0
0,14	25	0	0	0	1	0
0,15	25	0	0	0	0	0
0,18	25	0	1	0	0	0
0,14	29	0	0	0	0	0
0,15	28	0	1	0	0	0
0,16	30	0	1	0	0	0
0,14	24	0	1	0	0	0
0,2	29	0	1	0	0	0
0,12	22	0	1	0	0	0
0,17	25	0	1	0	0	0
0,16	27	0	1	0	1	0
0,15	27	0	1	0	1	0
0,15	20	0	1	0	0	0
0,14	25	0	0	0	0	0
0,15	29	0	0	0	0	0
0,16	22	0	0	0	1	0
0,16	26	0	1	0	0	0
0,11	29	0	1	0	0	0
0,18	31	0	0	0	0	0
0,11	22	0	1	0	0	0
0,12	31	0	0	0	0	0
0,2	32	0	5	0	1	0
0,14	23	0	0	0	1	0
0,14	26	0	1	0	0	0
0,15	21	0	0	0	0	0
0,2	30	0	0	0	0	0
0,11	22	0	1	0	1	0
0,14	35	0	0	0	0	0
0,17	27	0	0	0	1	0
0,17	30	0	0	0	0	0
0,13	24	0	0	0	0	0
0,12	25	0	0	0	0	0
0,16	30	0	0	0	0	0
0,16	29	0	0	0	0	0
0,15	26	0	0	0	0	0
0,15	27	0	0	0	0	0
0,15	24	1	5	0	1	0
0,16	24	0	1	0	0	0
0,16	32	0	1	0	0	0
0,15	27	0	1	0	0	0
0,11	20	0	0	0	0	0
0,19	30	0	1	0	0	0
0,14	20	0	1	0	0	0
0,2	26	0	1	0	0	0
0,14	28	0	0	0	0	0
0,16	28	0	1	0	1	0

0,08	22	0	0	0	1	0
0,15	23	0	1	0	1	0
0,16	26	0	0	0	0	0
0,15	26	0	0	0	1	0
0,15	26	0	1	0	1	0
0,16	25	0	1	0	0	0
0,15	29	0	0	0	1	0
0,15	24	0	0	0	1	0
0,18	23	0	0	0	1	0
0,16	24	0	0	0	1	0
0,16	22	0	0	0	1	0
0,13	27	0	0	0	0	0
0,13	22	0	0	0	0	0
0,16	31	0	1	0	0	0
0,13	23	0	0	0	0	0
0,15	24	0	1	0	0	0
0,15	22	0	1	0	1	0
0,16	28	0	0	0	0	0
0,14	23	0	1	0	0	0
0,11	20	0	2	0	1	0
0,15	21	0	1	0	1	0
0,15	34	0	0	0	1	0

ai	as	ti	spap	hb	hkt	probnp
1	0	1	26	13,9	39,6	87
1	0	0		14,7	42,1	116,4
0	0	0		15,2	44,3	104,9
0	0	0		15,3	42,8	167,5
0	0	0		15,8	44,4	5
2	1	0		14,7	41,2	55
0	0	0		15	42,6	13,1
0	0	0		14,8	43,2	65,7
0	0	0		16,5	47,3	21
0	0	0	24	14,5	41,4	15,2
0	0	1	38	14,6	42,7	26,2
0	0	0	24	15,2	44	57,3
0	0	0	30	16	45,6	40,7
0	0	0		13,9	40,1	63,9
0	0	0	12	15,5	44,2	24
0	0	0		14,7	41,3	18
0	0	1		15	40,5	11
0	0	0		14,5	42,6	102,9
0	0	0		14,9	43	32
0	0	0	26	12,9	38,1	49,3
2	0	1	28	15,2	43,3	119,2
0	0	0		15,3	44,4	18,4
0	0	1	38	13,6	39,5	300,9
0	0	1	30	14,3	43,4	81,4
0	0	1	28	15	43,7	47,8
0	0	1	36	14,9	42	93,7
0	0	0	32	15,2	42,33	65
0	0	0		15,6	47,3	89
0	0	0	30	14,4	42,1	16,5
0	0	1	30	14,2	39,5	20,7
0	0	1	36	13,8	41,6	250,3
0	0	0	24	13,6	41,1	22,4
0	0	1	26	12,5	35,8	109
0	0	1	36	15,2	44,1	37
0	0	1	19	15	42	16,8
0	0	0		16,9	47,9	27,9
0	0	0	36	15	42	54,1
1	0	1		15,1	45,2	37,5
0	0	0		16	46,5	6,1
0	0	0		14,4	41,9	58,9
0	0	0		15,7	47	14,7
0	0	0		14,1	40,4	11,6
0	0	0		13,8	39,7	33
0	0	0	34	15	42,3	5
0	0	0		14,1	40,9	116,9
0	0	0		14,3	41	26,4
2	0	0		15,3	42,6	77

0	0	0	28	15	42,4	48
0	0	1	46	13,4	39,9	33,5
0	0	0	30	12,5	37,2	19,7
0	0	0		13,5	38,8	55,5
0	0	0		15,1	42,8	26,5
0	0	0		15,1	41,8	5
0	0	0		14,2	41	77
0	0	0	30	13,4	38,8	103,8
0	0	0		14,2	41,1	12,2
0	0	0	32	13,5	40,9	84
0	1	0		14,4	40,3	15,1
1	0	0	30	17	46,2	27,1
0	0	1	34	13,1	37,9	76,3
0	0	0		16,2	43,2	43
0	0	0	28	14,5	42,4	68,4
0	0	0	36	14,7	42,3	37,5
1	0	0	26	15,2	43,4	94,4
0	0	0		13,9	40,9	17
0	0	0	36	12,9	37,9	73,9
0	0	1	20	14,8	42,1	33
0	0	1		15,4	43,5	5
0	0	0	0	15,8	46	55

hba1c	basalposteri	mitteposteri	apposterior	apantseptum	mitteantsept	basalantstep
5,1	-17	-16	-17	-18	-20	-16
5	-25	-22	-26	-24	-18	-11
5,3	-16	-22	-20	-19	-21	-23
5	-24	-15	-16	-27	-14	-6
5,3	-21	-18	-23	-25	-22	-15
5,2	-13	-22	-28	-30	-19	-8
5,5	-18	-20	-22	-21	-18	-18
5,3	-19	-17	-29	-36	-27	-15
5,5	-16	-15	-12	-14	-19	-12
5,2	-18	-20	-23	-28	-22	-17
5,6	-20	-22	-28	-26	-16	-13
5,2	-16	-22	-16	-19	-20	-26
5,6	-12	-14	-20	-21	-16	-11
5,6	-17	-18	-18	-16	-16	-15
5,3	-16	-15	-18	-16	-16	-15
5,5	-9	-18	-30	-24	-13	-18
5	-13	-14	-23	-31	-18	-11
5,6	-16	-15	-17	-19	-19	-18
5,2	-20	-15	-19	-23	-21	-18
5,2	-25	-26	-31	-34	-20	-16
5,6	-16	-16	-22	-26	-17	-11
5,5	-14	-7	-19	-20	-18	-16
5,1	-7	-8	-16	-23	-22	-18
5,2	-17	-11	-20	-27	-16	-12
5,2	-15	-19	-26	-21	-9	-6
5,2	-20	-26	-31	-32	-15	-11
5,33	-7	-17	-26	-32	-18	-14
5,7	-12	-15	-18	-23	-19	-14
5,5	-18	-16	-17	-23	-21	-31
5	-17	-16	-20	-17	-20	-26
5,9	-21	-16	-26	-32	-20	-17
5,6	-23	-4	-26	-26	-16	-10
5,2	-21	-20	-28	-31	-20	-14
5,4	-21	-19	-30	-33	-18	-14
5,4	-18	-17	-20	-28	-23	-14
5,5	-16	-19	-24	-24	-15	-11
5,4	-22	-22	-28	-28	-16	-16
5,9	-27	-16	-16	-31	-26	-21
5,4	-14	-18	-22	-20	-18	-16
5,2	-16	-16	-24	-26	-19	-19
5,2	-25	-21	-23	-33	-28	-23
5,3	-13	-16	-26	-29	-17	-12
5,4	-21	-17	-23	-24	-19	-13
5,1	-15	-14	-16	-20	-16	-13
5,6	-18	-15	-21	-18	-21	-29
4,9	-21	-20	-26	-31	-16	-15
5,6	-15	-16	-29	-36	-13	-15

5,3	-21	-17	-22	-21	-18	-15
5,4	-9	-16	-28	-31	-10	-3
5,7	-14	-21	-29	-20	-14	-11
4,9	-22	-16	-28	-32	-17	-12
5,6	-16	-21	-26	-25	-13	-9
5,4	-5	-12	-28	-36	-22	-7
5,4	-19	-21	-29	-31	-18	-14
5,4	-22	-22	-28	-23	-17	-14
5,2	-17	-17	-27	-28	-17	-12
5,4	-23	-19	-23	-29	-16	-10
5,5	-6	-12	-25	-31	-19	-14
4,8	-19	-18	-25	-25	-18	-14
5,3	-19	-20	-20	-23	-21	-15
5,5	-11	-13	-22	-29	-18	-12
5,4	-17	-19	-15	-11	-16	-17
5,4	-15	-22	-28	-24	-18	-15
5,4	-23	-15	-19	-35	-15	-7
5,7	-18	-20	-23	-24	-20	-19
5,4	-9	-14	-25	-29	-17	-14
5,1	-18	-15	-17	-18	-20	-20
5,1	-17	-19	-15	-20	-21	-16
5,4	-17	-15	-21	-17	-20	-28

basalinfsept	mitteinfsept	apinfseptum	aplateral	mittelateral	basallateral	basalinferior
-29	-20	-25	-25	-20	-25	-20
-14	-19	-20	-22	-27	-26	-14
-20	-18	-23	-22	-24	-21	-21
-9	-15	-28	-28	-23	-25	-19
-15	-19	-26	-21	-19	-25	-20
-13	-23	-32	-25	-22	-24	-20
-14	-19	-27	-24	-19	-23	-14
-15	-19	-24	-26	-22	-20	-23
-13	-17	-25	-15	-14	-19	-18
-17	-19	-26	-20	-16	-14	-15
-10	-19	-27	-24	-16	-18	-19
-24	-21	-23	-19	-19	-31	-21
-12	-18	-33	-26	-18	-15	-12
-11	-15	-20	-26	-22	-21	-17
-13	-15	-24	-27	-20	-20	-13
-17	-22	-31	-24	-19	-16	-25
-9	-16	-31	-27	-15	-18	-16
-12	-15	-24	-20	-18	-17	-11
-12	-20	-34	-29	-18	-19	-18
-16	-22	-30	-26	-19	-23	-19
-11	-19	-24	-20	-15	-15	-17
-10	-14	-16	-12	-17	-20	-12
-10	-19	-24	-19	-12	-9	-14
-13	-16	-24	-16	-5	-18	-14
-9	-17	-26	-18	-15	-16	-19
-14	-23	-30	-25	-14	-6	-31
-15	-22	-30	-31	-15	-17	-20
-9	-20	-23	-19	-13	-7	-17
-26	-24	-23	-25	-18	-20	-19
-36	-19	-22	-25	-20	-28	-18
-12	-22	-28	-19	-14	-14	-24
-9	-18	-28	-29	-10	-9	-17
-10	-20	-29	-23	-18	-15	-9
-22	-21	-30	-28	-20	-20	-20
-9	-17	-30	-24	-14	-4	-14
-15	-19	-21	-20	-19	-19	-17
-21	-21	-28	-26	-19	-22	-15
-11	-15	-26	-15	-17	-25	-17
-17	-19	-25	-15	-13	-13	-18
-10	-16	-36	-38	-18	-20	-14
-8	-18	-31	-30	-22	-22	-18
-10	-20	-30	-25	-13	-18	-21
-21	-23	-29	-32	-10	-12	-11
-11	-16	-23	-22	-14	-12	-17
-30	-20	-23	-23	-18	-30	-19
-4	-17	-32	-30	-22	-26	-12
-12	-19	-30	-14	-7	-10	-19

-10	-16	-24	-27	-21	-23	-11
-8	-11	-27	-27	-11	-9	-11
-12	-12	-16	-17	-26	-27	-12
-10	-21	-31	-26	-16	-20	-21
-11	-17	-25	-22	-19	-20	-16
-17	-13	-26	-26	-15	-10	-5
-6	-16	-32	-27	-22	-21	-14
-15	-17	-29	-31	-22	-21	-20
-10	-18	-27	-23	-15	-19	-15
-14	-20	-24	-15	-16	-17	-8
-11	-15	-22	-29	-21	-16	-14
-13	-21	-28	-25	-19	-16	-14
-10	-19	-30	-28	-21	-18	-18
-14	-26	-36	-19	-7	-14	-26
-9	-15	-18	-15	-18	-19	-14
-7	-13	-19	-21	-12	-7	-14
-7	-13	-20	-19	-29	-19	-8
-16	-19	-22	-9	-9	-14	-24
-11	-18	-18	-12	-11	-16	-19
-35	-24	-25	-23	-19	-30	-19
-32	-23	-24	-26	-19	-20	-21
-25	-21	-23	-23	-23	-16	-17

mitteinferior	apinferior	apanterior	mitteanterio	basalanterior	hrsperweek_	rechtsbasal
-23	-24	-22	-18	-17	1	-25
-22	-29	-28	-18	-6	0	-23
-18	-17	-20	-17	-17	1	-17
-20	-31	-26	-6	-6	0	-24
-22	-27	-20	-18	-14	1	-27
-23	-31	-26	-19	-12	0	-37
-16	-25	-23	-19	-21	1	-25
-22	-25	-19	-21	-21	1	-30
-21	-22	-15	-13	-17	0	-21
-19	-30	-22	-20	-18	0	-9
-19	-22	-15	-18	-16	1	-23
-25	-25	-21	-20	-16	0	-23
-13	-18	-15	-21	-17	0	-21
-16	-18	-17	-21	-21	1	-23
-13	-19	-20	-20	-20	0	-16
-23	-29	-21	-18	-15	0	-24
-16	-25	-26	-15	-13	1	-21
-17	-20	-14	-19	-27	0	-27
-19	-33	-33	-22	-21	1	-23
-24	-35	-35	-20	-20	1	-23
-16	-21	-24	-20	-15	1	-25
-13	-22	-18	-17	-10	1	-23
-17	-17	-12	-19	-15	0	-24
-16	-24	-24	-16	-18	1	-23
-19	-26	-22	-16	-13	1	-18
-33	-35	-32	-16	-14	0	-24
-26	-32	-34	-24	-20	1	-31
-18	-10	-10	-20	-20	1	-29
-19	-17	-18	-19	-20	1	-18
-25	-25	-21	-18	-17	0	-18
-29	-37	-30	-18	-9	1	-25
-26	-30	-34	-11	-8	1	-18
-17	-27	-20	-15	-16	1	-22
-21	-27	-23	-17	-18	1	-21
-20	-26	-24	-19	-13	0	-19
-18	-19	-19	-16	-23	1	-25
-19	-30	-30	-22	-19	1	-24
-18	-20	-21	-18	-13	0	-24
-17	-20	-4	-18	-13	1	-26
-20	-26	-27	-31	-30	0	-26
-24	-27	-19	-23	-24	0	-25
-23	-34	-28	-19	-15	1	-19
-17	-23	-26	-24	-18	0	-25
-15	-19	-18	-17	-13	1	-18
-20	-25	-23	-18	-20	1	-26
-18	-23	-24	-22	-18	1	-29
-18	-28	-31	-15	-7	0	-24

-16	-25	-24	-22	-19	1	-19
-10	-33	-19	-11	-11	1	-24
-14	-23	-20	-19	-24	1	-29
-23	-28	-16	-18	-19	1	-20
-19	-25	-23	-23	-16	0	-18
-11	-23	-24	-21	-13	1	-38
-17	-28	-33	-26	-24	0	-28
-20	-26	-18	-19	-22	1	-19
-18	-24	-21	-20	-15	1	-34
-8	-14	-12	-10	-9	1	-20
-13	-25	-32	-23	-20	1	-15
-18	-20	-21	-22	-21	1	-21
-18	-20	-16	-20	-17	1	-22
-22	-17	-11	-13	-17	1	-27
-15	-17	-15	-19	-22	1	-19
-15	-17	-15	-13	-11	1	-20
-15	-24	-30	-16	-10	0	-23
-26	-18	-12	-17	-18	0	-22
-17	-22	-16	-16	-17	1	-26
-23	-26	-23	-19	-17	0	-26
-22	-23	-21	-18	-18	1	-25
-19	-24	-21	-19	-19	1	-24

rechtsmitte	rechtsapical	bsa	rwt	lavolume	lavoulume_ii	lv_bsa
-26	-27	0,348837	0,595	107	306,7333	27,49534
-26	-29	0,357143	0,5	105	294	31,53886
-23	-30	0,298508	0,461	109	365,1499	27,00532
-27	-33	0,186335	0,64	121	649,3667	21,51946
-27	-28	0,229183	0,545	73	318,5233	26,24778
-39	-40	0,40655	0,605	65	159,8819	29,93151
-27	-29	0,269139	0,462	67	248,9422	22,89517
-32	-35	0,278616	0,619	89	319,4359	27,93938
-19	-24	0,27907	0,591	67	240,0833	25,00762
-16	-26	0,281955	0,619	64	226,9866	26,90969
-26	-28	0,266667	0,762	79	296,2499	28,3154
-22	-26	0,206186	0,56	75	280,3454	22,15258
-21	-19	0,265252	0,462	77	290,29	25,42678
-22	-21	0,222662	0,509	88	395,2179	24,16942
-18	-18	0,227359	0,651	67	294,6884	21,09789
-25	-27	0,25	0,591	69	276	26,54751
-22	-24	0,27178	0,565	77	283,3172	27,03305
-27	-28	0,199446	0,56	68	340,9444	25,07222
-25	-27	0,324675	0,489	63	194,04	28,5725
-26	-29	0,341556	0,512	62	181,5222	26,18209
-26	-27	0,305111	0,533	59	193,3725	27,12668
-25	-27	0,208213	0,489	113	542,714	23,09508
-25	-26	0,255682	0,436	135	528	29,02008
-27	-28	0,363636	0,622	58	159,5	27,13039
-19	-21	0,297275	0,609	78	262,3833	27,28058
-27	-29	0,382653	0,6	64	167,2533	26,96232
-38	-44	0,225564	0,357	76	336,9333	25,47489
-30	-32	0,420168	0,578	68	161,84	28,85394
-19	-21	0,211528	0,571	64	302,56	22,74822
-18	-20	0,263717	0,609	65	246,4764	25,32832
-26	-27	0,291309	0,531	62	212,8322	29,52123
-19	-21	0,24948	0,52	88	352,7334	27,29962
-23	-26	0,35475	0,408	66	186,0467	27,10324
-22	-22	0,337584	0,522	90	266,6	28,26866
-27	-34	0,246322	0,48	104	422,2111	23,69648
-26	-27	0,256009	0,75	82	320,3011	24,87618
-26	-27	0,268777	0,462	80	297,6444	27,84755
-24	-26	0,196721	0,578	100	508,3334	25,20836
-28	-30	0,23678	0,591	81	342,09	22,96656
-26	-28	0,184332	0,542	114	618,45	20,47996
-26	-28	0,24948	0,5	75	300,6251	24,81784
-27	-31	0,318134	0,596	55	172,8833	26,82001
-26	-27	0,27027	0,591	63	233,1	25,37077
-19	-21	0,180451	0,533	78	432,2501	22,7409
-25	-28	0,307298	0,51	75	283,217	26,94103
-27	-28	0,253593	0,56	76	299,6933	27,12286
-25	-25	0,290089	0,489	80	275,7778	28,226

-20	-21	0,295858	0,458	72	243,36	27,41697
-22	-21	0,276646	0,775	104	375,9312	26,64571
-29	-28	0,290089	0,5	81	279,225	27,68319
-22	-24	0,332226	0,511	99	297,99	28,65008
-20	-21	0,235294	0,55	61	259,25	24,89592
-39	-36	0,275503	0,522	52	188,7455	24,05253
-33	-37	0,25641	0,6	54	210,6	21,77155
-21	-26	0,380791	0,536	57	149,6883	29,61979
-37	-39	0,238095	0,625	65	273,0001	26,59184
-18	-15	0,363196	0,591	75	206,5	30,56627
-19	-23	0,237014	0,423	90	379,7251	25,3368
-21	-20	0,225975	0,444	67	296,4936	23,55161
-20	-18	0,304311	0,619	103	338,4694	26,08237
-29	-34	0,237938	0,608	57	239,5583	22,63779
-21	-26	0,239282	0,5	62	259,1084	26,18942
-21	-21	0,285714	0,634	43	150,5	28,87078
-26	-28	0,299003	0,622	75	250,8333	24,65593
-23	-25	0,255102	0,444	59	231,28	26,82064
-29	-31	0,447205	0,636	58	129,6944	28,51616
-26	-27	0,218579	0,52	78	356,8501	23,53822
-26	-27	0,239282	0,445	71	296,7209	24,707
-22	-21	0,238095	0,44	79	331,8	25,51207

rv_bsa	la_bsa	ra_bsa	ivs_bsa	lav_bsa	EF	heartrate
21,06026	27,49534	27,49534	5,850073	62,59578	60	47
19,04233	32,13393	32,72901	5,355656	62,48265	62	59
19,98394	25,385	24,84489	5,94117	58,8716	63	76
16,68856	27,66788	27,66788	5,709245	53,13989	67	65
15,27143	28,63394	29,5884	4,772323	34,83796	63	65
15,28417	28,02099	26,11047	4,457884	41,39464	63	83
17,69172	28,09862	28,09862	5,723793	34,8631	71	63
18,45053	31,62948	32,15664	5,798738	46,91706	62	68
15,96231	25,5397	25,5397	5,852848	35,64916	58	69
15,06943	28,52427	28,52427	4,843744	34,4444	57	68
20,0782	28,3154	26,77093	6,692732	40,67122	60	45
17,53746	23,99863	23,99863	5,999658	38,6453	64	58
14,01067	29,57809	29,57809	6,226966	39,95637	59	61
16,91859	26,10297	27,55314	5,800661	42,53818	62	69
19,1799	24,93386	25,41336	5,274471	32,12632	67	64
19,03406	24,54392	25,04482	5,50986	34,56185	57	51
18,71519	25,99332	28,59265	6,758263	40,02971	61	62
15,67014	23,72906	25,07222	2,686309	30,44484	60	61
15,42915	27,4296	27,4296	5,7145	36,00135	61	58
21,52749	28,50938	28,50938	4,654593	36,0731	60	80
17,71539	30,44832	29,34111	6,64327	32,66274	59	61
16,62845	23,09508	23,09508	5,542818	52,19487	55	63
16,8011	29,5292	30,03833	6,10949	68,73177	59	54
21,10142	34,36516	34,36516	6,028976	34,96806	57	59
19,64202	27,28058	27,28058	6,547339	42,5577	57	53
18,99618	30,02622	30,02622	6,1278	39,21791	64	60
21,22908	24,05962	24,05962	4,717573	35,85355	63	54
18,3616	27,5424	27,5424	6,557714	44,59245	60	67
14,39173	25,53372	25,53372	5,106743	29,71196	58	72
17,22326	26,84801	28,36771	5,572229	32,92681	64	78
20,22751	30,06792	28,97454	3,826827	33,89475	63	40
13,89799	27,79598	26,30691	4,963568	43,67939	56	82
15,90843	28,87085	28,28165	6,481211	38,88726	56	62
14,42278	28,26866	28,26866	5,192202	51,92202	58	49
20,73442	26,65854	25,67119	5,92412	51,34238	64	62
17,26102	24,87618	24,87618	5,076772	41,62953	56	51
19,08073		27,26465	5,156953	41,25563	61	56
15,30508	33,7612	33,7612	7,652539	45,01493	63	59
14,6595	23,94386	23,94386	5,863801	39,58066	56	57
16,55827	27,01612	27,01612	5,664671	49,6748	69	47
17,86884	30,27776	31,76683	5,459924	37,22676	69	57
17,68979	24,53745	24,53745	6,277023	31,38511	59	55
17,60421	25,88854	25,37077	5,69548	32,61956	61	70
13,30128	23,16998	23,16998	4,71981	33,46775	64	48
15,15433	29,74739	29,74739	5,051444	37,2324	55	71
18,58418	27,12286	27,12286	6,027302	38,17291	59	64
14,113	31,48285	31,48285	7,0565	43,42461	57	68

20,28856	31,25535	31,80369	6,031734	39,48044	67	40
19,71782	34,10651	32,50776	9,059541	55,42307	63	58
20,08388	29,31161	30,39723	5,428077	43,96742	54	65
16,04404	31,51509	31,51509	6,303018	56,72716	58	57
16,59728	26,84855	26,84855	5,369709	29,77748	55	67
16,7322	24,57542	23,00677	6,274574	27,18982	66	69
18,22734	27,84733	26,8347	5,063151	27,34102	64	59
19,12945	29,00271	29,00271	4,936632	35,1735	56	57
17,40557	23,20742	24,65788	4,834879	31,42671	61	60
19,17884	31,16561	29,96694	5,993387	44,95041	57	64
18,51536	27,28579	26,79854	5,359708	43,85216	59	71
13,93871	26,43548	26,43548	6,248386	32,20322	59	51
18,31315	29,41203	29,41203	5,54944	57,15923	56	47
13,77952	23,62204	23,62204	6,397635	28,05117	57	69
18,77732	25,20114	25,20114	5,43554	30,63668	58	54
17,64326	27,26685	27,26685	5,881085	22,9897	58	69
20,27265	29,03921	26,84757	6,574915	41,09322	58	53
19,5997	26,82064	26,82064	3,61047	30,43111	64	62
20,55816	31,832	31,832	5,9685	38,46367	54	58
17,41828	24,95051	24,95051	5,178408	36,71962	55	60
18,77732	25,69528	27,1777	5,43554	35,08394	56	55
14,7185	26,49331	26,49331	5,396785	38,75872	56	68

rrsys	rrdia	globalRVstrain	globalLVstrain	basalLVstrain	midLVstrain	apLVstrain
112	78	-26	-21	-21	-20	-22
129	81	-26	-21	-16	-21	-25
110	89	-23	-20	-20	-20	-20
149	90	-28	-19	-15	-16	-26
139	89	-27	-21	-18	-20	-24
137	91	-39	-22	-15	-21	-29
126	82	-27	-20	-18	-19	-24
117	74	-32	-22	-19	-21	-27
129	82	-21	-17	-16	-17	-17
116	80	-17	-20	-17	-19	-25
138	90	-26	-19	-16	-18	-24
111	82	-24	-21	-22	-21	-21
129	81	-20	-17	-13	-17	-22
119	77	-22	-18	-17	-18	-19
126	84	-17	-18	-16	-17	-21
110	77	-25	-21	-17	-19	-27
131	79	-22	-19	-13	-16	-27
119	82	-27	-18	-17	-17	-19
127	84	-25	-22	-18	-19	-29
109	81	-26	-25	-20	-22	-32
141	87	-26	-18	-14	-17	-23
117	77	-25	-15	-14	-14	-18
131	85	-25	-16	-12	-16	-19
126	89	-26	-17	-15	-13	-23
129	78	-19	-17	-13	-16	-23
116	72	-27	-23	-16	-21	-31
146	90	-38	-22	-16	-20	-31
139	88	-30	-16	-13	-18	-17
118	77	-19	-21	-22	-20	-21
127	82	-19	-22	-24	-20	-22
136	89	-26	-22	-16	-20	-29
110	78	-19	-19	-13	-14	-29
129	81	-24	-20	-14	-18	-26
116	87	-22	-22	-19	-19	-29
126	84	-27	-19	-12	-18	-25
130	85	-26	-19	-17	-18	-21
129	70	-26	-22	-19	-20	-28
132	90	-25	-20	-19	-18	-22
121	83	-28	-17	-15	-17	-18
117	86	-27	-23	-18	-20	-30
126	77	-26	-23	-20	-23	-27
128	90	-26	-21	-15	-18	-29
130	88	-26	-20	-16	-18	-26
124	80	-19	-16	-14	-15	-20
135	88	-26	-22	-24	-19	-22
115	78	-28	-21	-16	-19	-28
129	75	-25	-19	-13	-15	-28

117	79	-20	-20	-17	-18	-24
146	91	-22	-16	-9	-12	-28
121	76	-29	-18	-17	-18	-21
128	81	-22	-21	-17	-19	-27
125	83	-20	-19	-15	-19	-24
132	89	-38	-17	-10	-16	-27
122	81	-33	-22	-16	-20	-30
119	80	-22	-21	-19	-20	-26
129	84	-37	-19	-15	-18	-25
132	92	-18	-16	-14	-15	-20
123	77	-19	-19	-14	-17	-27
136	82	-21	-20	-16	-19	-24
120	76	-20	-20	-16	-20	-23
131	86	-30	-18	-16	-17	-22
128	81	-22	-16	-16	-17	-15
137	89	-21	-16	-12	-16	-21
133	85	-26	-18	-12	-17	-25
136	88	-23	-18	-18	-19	-18
129	84	-29	-17	-14	-16	-20
136	87	-26	-22	-23	-20	-22
118	83	-26	-21	-21	-20	-22
134	92	-22	-20	-20	-20	-22
